# Comparison of Fruit and Vegetable Intake Among Urban Low-Income US Adults Receiving a Produce Voucher in 2 Cities

**DOI:** 10.1001/jamanetworkopen.2021.1757

**Published:** 2021-03-22

**Authors:** Sanjay Basu, Melissa Akers, Seth A. Berkowitz, Kevin Josey, Dean Schillinger, Hilary Seligman

**Affiliations:** 1 Center for Primary Care, Harvard Medical School, Boston, Massachusetts; 2 Research and Population Health, Collective Health, San Francisco, California; 3 Ariadne Labs, Brigham and Women’s Hospital and Harvard T.H. Chan School of Public Health, Boston, Massachusetts; 4 School of Public Health, Imperial College, London, United Kingdom; 5 Center for Vulnerable Populations, Division of General Internal Medicine, San Francisco General Hospital/University of California San Francisco, San Francisco; 6 Division of General Medicine and Clinical Epidemiology, Department of Medicine, University of North Carolina at Chapel Hill, Chapel Hill; 7 Department of Biostatistics, Harvard T.H. Chan School of Public Health, Boston, Massachusetts

## Abstract

**Importance:**

Fruit and vegetable vouchers have been implemented by cities and counties across the US to increase fruit and vegetable intake and thereby improve overall nutritional quality.

**Objective:**

To determine whether and why use of fruit and vegetable vouchers are associated with varied nutritional intake across different populations and environments.

**Design, Setting, and Participants:**

In a population-based pre-post cohort study of 671 adult participants with low income before and during (6 months after initiation) participation in a 6-month program, fruit and vegetable vouchers were distributed for redemption at local San Francisco and Los Angeles neighborhood grocery and corner stores between 2017 and 2019. A transportability analysis was performed to identify factors that may explain variation in voucher use between cities.

**Exposure:**

Receipt of $20 per month in produce vouchers for 6 months from 2017 to 2019.

**Main Outcomes and Measures:**

Change in total fruits and vegetables (as defined by the US Department of Agriculture) consumed per person per day (change in cup-equivalents between month 6 and month 0).

**Results:**

A total of 671 adults (median age, 54.9 years [interquartile range, 45.0-65.0 years]; 61.7% female; 30.9% Black; 19.7% Hispanic) were enrolled. An increase in fruit and vegetable intake of 0.22 cup-equivalents per day overall (95% CI, 0.14-0.31 cup-equivalents; *P* < .001) was observed. However, the observed increase was larger in Los Angeles compared with San Francisco (0.64 cup-equivalents per day; 95% CI, 0.41-0.88 cup-equivalents vs 0.10 cup-equivalents per day; 95% CI, 0.01-0.19 cup-equivalents). When the concurrently sampled San Francisco group (n = 157) was weighted in transportability analysis to demographically match the Los Angeles group (n = 155) in observed covariates, the weighted San Francisco group had an estimated increase of 0.53 fruit and vegetable cup-equivalents per day (95% CI, 0.27- 0.79 cup-equivalents, *P* = .03), with income being the variable needed to allow the 95% confidence intervals to overlap between the weighted San Francisco and unweighted Los Angeles populations.

**Conclusions and Relevance:**

In this study, the use of fruit and vegetable vouchers appeared to be associated with greater benefit among those with lower incomes, suggesting that further investigation of flat-rate rather than income-scaled benefits is warranted.

## Key Points

**Question:**

Are produce food vouchers associated with different changes in diet among different populations, and what characteristics are associated with potential differences?

**Findings:**

In this pre-post cohort study of 671 adult users of community-based produce voucher programs in 2 cities, use of vouchers was associated with less of an improvement in fruit and vegetable intake and a lower composite nutrition index in San Francisco than in Los Angeles. In a statistical transportability analysis conducted to assess reasons for the difference, lower income of the Los Angeles population appeared to be a primary factor in the difference.

**Meaning:**

In this study, use of statistical transportability methods helped to identify that produce vouchers were associated with the greatest dietary improvements in the lowest income populations, concordant with principles of flat-rate rather than income-scaled benefit programs.

## Teaser

This pre-post cohort study assesses the association between food vouchers for produce and different changes in diet among adults in Los Angeles and San Francisco, California.

## Introduction

Fruit and vegetable vouchers have been implemented by cities and counties across the US to supplement federal nutrition programs and reduce the risk of diet-sensitive chronic disease.^1,2,3,4,5^ Fruit and vegetable vouchers have been tested in a previous randomized clinical trial in San Francisco, which reported that the vouchers improved fruit and vegetable intake and overall dietary quality.^6^ However, voucher impact in a trial based in 1 city may not be applicable to other diverse populations and environments.

In the present study, we sought to determine whether the same fruit and vegetable voucher program would have similar associations with fruit and vegetable consumption and overall dietary quality indexes among a different population of low-income adults in a different city (Los Angeles). In both San Francisco and Los Angeles, we distributed fruit and vegetable vouchers ($20/mo for 6 months) for redemption at local grocery and corner stores. We performed 24-hour dietary recalls before and during voucher receipt to assess changes in diet. Then we performed a transportability analysis, a statistical analysis that reweights participants in 1 city to resemble participants in another city by matching the sample moments (eg, means) of primary covariates associated with the intervention. We performed this step to help determine whether and why intervention outcomes may vary across populations. We sought to answer the question of whether food vouchers to improve nutrition are associated with different changes in diet among different populations. We hypothesized that differences in measured demographic features, rather than unmeasured neighborhood or contextual factors, were explanatory variables.

## Methods

### Design

We performed a pre-post comparison of low-income adults living in San Francisco and Los Angeles before and during (6 months after initiation) participation in a 6-month program that provided $20 per month in fruit and vegetable vouchers during the period from 2017 to 2019. Written informed consent was obtained from all participants prior to enrollment. The study was approved by the Institutional Review Boards of the University of California San Francisco and Stanford University.

### Participants

Inclusion criteria to the study were age 21 years or older, household income less than 250% of the federal poverty level, regular access to a telephone; English fluency sufficient to provide informed consent and complete detailed dietary recalls, and residency in the City of San Francisco or Los Angeles as defined by official municipal boundaries. Exclusion criteria were participating in a diet, including any other dietary or nutrition study (eg, an experimental weight loss research study); current diagnosis of cancer or congestive heart failure; currently pregnant; or planning to move out of the current city of residence within the next year. We recruited participants through community ads, public information sessions, and outreach events at community partner sites such as food banks, community clinics, and public housing sites in the Mission, Bayview, and Tenderloin neighborhoods of San Francisco, and in the Westlake and MacArthur Park neighborhoods of Los Angeles. The neighborhoods were selected because their populations had low average household incomes and a high prevalence of chronic disease associated with poor nutrition.^7^

### Intervention

Each participant received fruit and vegetable vouchers and information on redemption locations (names, addresses, and hours of operation) printed in English and Spanish. Vouchers were check sized, printed on secure paper, and included a customer signature line and date line to use during redemption, a start date and expiration date, and a clearly-printed redemption value ($5 for each voucher). The design is similar to the design of California Women, Infants and Children (WIC) Program vouchers familiar to cashiers.^8^ Similar to WIC rules, qualified fruits and vegetables included fresh or frozen fruits, vegetables, or herbs without added sugars or fats.^9^ The usage rules were printed on the front of the voucher and reviewed during in-person orientation sessions conducted at community sites. No additional nutrition education, food preparation, or cooking training was provided. All vouchers had the same individual value ($5), and participants received 4 vouchers for each calendar month (a total of $20) for a total of 6 months. Vouchers could not be redeemed for cash, and unredeemed portions of partially used $5 vouchers were not compensated (ie, participants were not provided change for the unused value). Vouchers for the next month were mailed to each participant during the last week of the previous month. To redeem, participants presented the voucher to a cashier and signed and dated the voucher at the time of redemption. The cashier processed the voucher just as they would WIC vouchers or manufacturer coupons, and the vendor then submitted the redeemed voucher for payment by research staff. Random observations of voucher use, including secret shoppers, were performed across store sites to ensure proper cashier acceptance of vouchers and did not reveal any acceptance problems. Participants were given contact information for study staff to resolve any difficulties in using the vouchers, and motivational interviewing techniques during the baseline assessment and throughout study participation were used to maintain retention.

### Study Settings

Participating vendors were chosen based on the following criteria: proximity to the low-income neighborhoods identified in study recruitment, proximity or easy access to public transportation, and the quantity, quality, and price of fruits and vegetables available. Vouchers were accepted at 29 vendors in San Francisco (including 23 grocery stores, 4 produce markets, and 2 farmers markets) in and around the Mission, Bayview, and Tenderloin neighborhoods, and at 5 vendors in Los Angeles (including 3 grocery stores and 2 produce markets) in the Westlake and MacArthur Park neighborhoods.

### Outcomes

Following informed consent, participants completed a baseline demographic survey and 2 telephone-based 24-hour dietary recalls in either English and Spanish with registered dieticians who entered the information into the Nutritional Dietary System for Research.^10^ Recalls were performed both before vouchers were received in month 0 and during the last month (month 6) of voucher receipt in both the San Francisco and Los Angeles samples. Two recalls were performed in month 0 and 2 in month 6 during any 2 days of the first week of the month. Individuals who completed both month 0 recalls and at least 1 month 6 recall were considered as having met all study requirements for inclusion in the final analysis of month 0 to month 6 outcome differences. The primary outcome metric was change in total fruits and vegetables (as defined by the US Department of Agriculture^11,12^) consumed per person per day (change in cup-equivalents between month 6 and month 0). Secondary outcomes were changes in overall dietary quality as measured by change in the 2015 Healthy Eating Index (HEI)^13^ total score (range: 0-100, higher scores indicate better diet quality), and differences in voucher use (% of distributed vouchers redeemed in stores). The primary outcome and the HEI outcome were estimated from 24-hour dietary recalls; the voucher use outcome was determined from accounting records. NCI MIXTRAN and DISTRIB macros were used to estimate usual intake from the recalls.^14^

### Statistical Analysis

Two statistical analyses were performed: a standard regression and a transportability analysis. In the standard regression, we calculated the change in fruit and vegetable consumption (primary outcome) and change in HEI score (secondary outcome) between months 0 and month 6, with an ordinary least squares regression coefficient estimating the association between study site (San Francisco vs Los Angeles) and the outcomes, after adjustment for age, sex, race/ethnicity, income, educational level, household size, and WIC/SNAP (Supplemental Nutrition Assistance Program) participation status. Data were not missing for the baseline demographic survey; for dietary data, we did not impute dietary recall data because we could not tell the difference between data missing due to lack of recall vs dietary components truly not consumed. A power calculation was performed to detect a minimum 124 people per site to detect with 80% power (2-tailed significance threshold of *P* < .05) and a Cohen *d* = 0.4 moderate effect size assuming 20% attrition.

In the transportability analysis, we studied whether the differences in voucher outcomes between study sites could be associated with measured differences between the groups (demographic characteristics and income) or with unmeasured confounders (eg, differences in the food purchasing environment, including store availability or fruit and vegetable prices). While a standard regression seeks to adjust for covariates, estimating the mean association between the intervention in isolation from these factors, transportability analysis attempts to estimate how a treatment association may be different in a different population, taking into account differences in distributions of characteristics that might modify the association between treatment and the outcomes. The transportability analysis involved statistically weighting the San Francisco population to match the measured characteristics of the Los Angeles population (eAppendix in the Supplement). The analysis then involved fitting a generalized linear model that included potential modifiers (age, sex, race/ethnicity, income, educational level, household size, and WIC/SNAP participation). This analysis helped in assessing whether results in the San Francisco group (weighted to resemble the Los Angeles group) were similar to those observed in the unweighted Los Angeles group. If the results were similar, then the similarity would suggest that differences between the sites could be explained by differences in the measured modifiers. If results were not similar, this finding would suggest that unmeasured characteristics would have to be the explanatory factors. For these transportability analyses, we used a previously validated transportability method called *entropy balancing*, a method that reweights a survey sample to known characteristics from a target population.^15^

**Figure 1.**
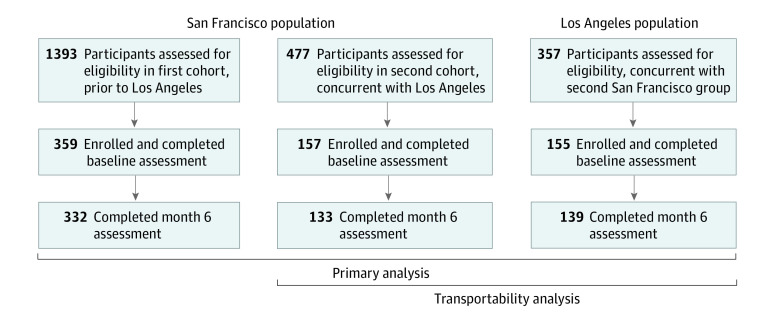
Distribution of Recruited Populations by Study Site

We note that the San Francisco voucher program was established earlier than the Los Angeles program, and the San Francisco program was tested in an initial group of 359 people in 2017 and 2018 (hereafter termed SF1; vouchers redeemed from February 10, 2017, through April 30, 2018), followed by a second San Francisco group of 157 people in 2018 and 2019 (hereafter termed SF2; vouchers redeemed from June 1, 2018, through September 30, 2019) who were tested concurrently with the Los Angeles group of 155 people (Figure 1). Hence, while we performed an overall analysis of voucher impact including the SF1, SF2, and Los Angeles groups, we focused the transportability analysis on the SF2 and Los Angeles groups to reduce confounding by secular trends or seasonal variations in food consumption. We explored whether the voucher use rate could help explain differences in outcomes among the 3 groups. Additionally, because the SF2 cohort was enrolled after the implementation of a tax on sugar-sweetened beverages, we studied whether exposure of the SF2 group to a sugar-sweetened beverage tax could help explain differences in outcomes among the 3 groups (eAppendix in the Supplement). Analyses were performed in R version 4.0.3 (R Project for Statistical Computing). This study followed the Strengthening the Reporting of Observational Studies in Epidemiology (STROBE) reporting guideline for cohort studies.

## Results

### Demographic Characteristics of the Study Populations

A total of 671 adults were enrolled in the study (Table 1). Median (interquartile range [IQR]) age was 54.9 (45.0-65.0) years; 67.1% were female, 30.9% were Black, and 19.7% were Hispanic. The median monthly income was $1000.00 (IQR, $845.00-$1467.00) in San Francisco (equivalent to approximately $10 000 to $12 000 dollars per year) and the median monthly income was $916.67 (IQR, $668.34-$1200.00) in Los Angeles, both values below the $12 760 median federal poverty level for households of the size observed in the study. Most participants reported living alone in their household (median household size for Los Angeles and San Francisco was 1.00 person [IQR, 1.00-2.00 people]); some individuals were also participants in SNAP (28.1% participants in the San Francisco population, 37.4% in Los Angeles). Of 2227 people assessed for eligibility, 671 met the inclusion/exclusion criteria and agreed to enroll (demographic characteristics of those not agreeing to participate were not recorded). Of the 671 participants enrolled, 604 completed month 6 recalls (10.0% loss to follow-up). A subgroup analysis showed no substantive differences between the earlier vs later San Francisco participants (eTable 1 in the Supplement).

**Table 1.** Descriptive Statistics of the Study Participants

Characteristic	No. (%)		*P* value for difference
	Los Angeles (n = 155)	San Francisco (n = 516)	
Age, median (IQR), y	58.0 (46.5-67.0)	54.0 (42.0-62.0)	.001
Male sex	65 (41.9)	192 (37.2)	
Female sex	90 (58.1)	324 (62.8)	.16
Race/ethnicity			
Black	71 (45.8)	136 (26.4)	<.001
Hispanic	52 (33.5)	80 (15.5)	<.001
Educational level			
Never attended school	1 (0.6)	1 (0.2)	<.001
Elementary or middle school	9 (5.8)	7 (1.4)	
Some high school	19 (12.3)	33 (6.4)	
High school graduate	37 (23.9)	87 (16.9)	
Some college or technical school	59 (38.1)	211 (40.9)	
College graduate	26 (16.8)	145 (28.1)	
Other	4 (2.6)	32 (6.2)	
Household income, median (IQR), $/mo	916.67 (668.34-1200.00)	1000.00 (845.00-1467.00)	.002
Household size, people, median (IQR)	1.00 (1.00-2.00)	1.00 (1.00-2.00)	.91
SNAP participant	58 (37.4)	145 (28.1)	.03
WIC Program participant	3 (1.9)	15 (2.9)	.71

Abbreviations: IQR, interquartile range; SNAP, Supplemental Nutrition Assistance Program; WIC, Women, Infants, and Children.

### Primary and Secondary Outcomes

Table 2 disaggregates the primary and secondary outcomes by time and study site. The primary outcome was a within-person change from month 0 to month 6 of 0.22 cup-equivalents of fruits and vegetables among the overall study population across all sites and time frames (95% CI, 0.14-0.31 cup-equivalents; *P* < .001). The baseline fruit and vegetable intake among participants was a mean (SD) of 1.10 (0.88) cup-equivalents (median: 0.89; IQR, 0.47-1.55 cup-equivalents), and the month 6 intake of a mean (SD) of 1.33 (1.12) (median: 1.11; IQR, 0.61-1.75 cup-equivalents), with 604 of the 671 participants completing month 6 dietary recalls (90.0% overall, with 90.1% completion from San Francisco and 89.7% from Los Angeles). Recalls were performed on either weekdays or weekends, with 27.5% of recalls in San Francisco performing a recall of a weekend day and 30.9% of recalls in Los Angeles performing a recall of a weekend day (*P* = .94 for differences in days of the week between the cohorts).

**Table 2.** Primary and Secondary Outcomes by Study Site and Time of Evaluation

Outcome	Month 0, baseline, mean (SD)	No. (%)	Month 6, during voucher, mean (SD)	No. (%)	Within-person change, month 0 to month 6 (95% CI)	*P* value for within-person change
**Primary**						
Fruit and vegetable intake (cup-equivalents)						
Overall population	1.10 (0.88)	671 (100)	1.33 (1.12)	604 (100)	0.22 (0.14 to 0.31)	<.001
San Francisco	1.14 (0.87)	516 (76.9)	1.26 (1.06)	465 (77.0)	0.10 (0.01 to 0.19)	.02
Los Angeles	0.97 (0.91)	155 (23.1)	1.58 (1.28)	139 (23.0)	0.64 (0.41 to 0.88)	<.001
**Secondary**						
Healthy Eating Index^a^						
Overall population	61.9 (13.0)	671 (100)	64.2 (12.7)	604 (100)	2.0 (1.0 to 3.0)	<.001
San Francisco	62.7 (12.5)	516 (76.9)	63.7 (12.8)	465 (77.0)	0.6 (−0.5 to 1.7)	.27
Los Angeles	59.5 (14.2)	155 (23.1)	66.1 (12.2)	139 (23.0)	6.8 (4.3 to 9.2)	<.001

^a^ Healthy Eating Index total score (range: 0-100, higher scores indicate better diet quality).

The primary outcome substantially varied between study sites. Among the San Francisco participants (overall), baseline mean (SD) fruit and vegetable intake was 1.14 (0.87) cup-equivalents of fruits and vegetables (median: 0.94; IQR, 0.51-1.61 cup-equivalents), and month 6 intake was 1.26 (1.06) cup-equivalents (median: 1.05; IQR, 0.59-1.67 cup-equivalents), with mean a (SD) within-person increase of 0.10 (0.04) cup-equivalents (95% CI, 0.01-0.19 cup-equivalents; *P* = .02). Among the Los Angeles participants, baseline mean (SD) intake was 0.97 (0.91) cup-equivalents (median: 0.77; IQR, 0.31-1.34 cup-equivalents), and month 6 intake was 1.58 (1.28) cup-equivalents (median: 1.33; IQR, 0.71-2.12 cup-equivalents), for a within-person increase of 0.64 cup-equivalents (95% CI, 0.41-0.88 cup-equivalents; *P* < .001). eTable 2 in the Supplement further disaggregates the San Francisco participants by earlier (SF1) vs later (SF2) groups, which had similar results. When comparing the concurrently sampled the San Francisco group with the Los Angeles group, controlling for demographic covariates, use of the voucher was associated with a greater change in outcomes in Los Angeles than in San Francisco (a difference of more than 0.50 cup-equivalents of fruits and vegetables in Los Angeles than in San Francisco; 95% CI, 0.13-0.87; *P* = .01). A fully adjusted model is available in eTable 3 in the Supplement.

The secondary outcome of HEI total score showed improvement consistent with the primary outcome of fruit and vegetable intake. Among the San Francisco participants (overall), the mean (SD) baseline HEI score was 62.7 (12.5) (on a scale of 0 to 100, in which a greater score indicates better diet quality; median: 62.9; IQR, 53.4-71.3; national mean benchmark of 59^13^), and the month 6 HEI score was 63.7 (12.8) (median: 64.5; IQR, 54.0-73.0), for a within-person improvement in score of 0.6 (95% CI, −0.5 to 1.7; *P* = .27). In the Los Angeles participants, mean (SD) baseline HEI total score was 59.5 (14.2) (median: 58.4; IQR, 49.6-70.2), and month 6 HEI total score was 66.1 (12.2) (median: 66.2; IQR, 57.9-75.8), for a within-person improvement in score of 6.8 (95% CI, 4.3-9.2; *P* < .001). eTable 2 in the Supplement further disaggregates the San Francisco participants by SF1 vs SF2, which had similar results. When comparing the concurrently sampled San Francisco group with the Los Angeles group, controlling for demographic covariates, use of the voucher was associated with a greater change in outcomes in Los Angeles than in San Francisco (6.5 greater improvement in HEI total score in Los Angeles than in San Francisco; 95% CI, 2.6-10.4; *P* = .001) (eTable 4 in the Supplement). The HEI subcomponents are shown in eTable 5 in the Supplement, which indicate that the improvement in HEI total score in Los Angeles may have been associated with increased total and whole fruit and green bean consumption.

The voucher redemption rate was 75.6% overall (mean within-person proportion of mailed vouchers that were used per accounting logs), with a rate of 74.6% in San Francisco (75.2% in SF1 and 73.4% in SF2) and 79.0% in Los Angeles. Voucher redemption did not explain the differential outcome across locations in a standard regression analysis including an interaction term between study site and voucher redemption rate (eTable 6 in the Supplement). Notably, redemption timing (proportion of vouchers redeemed in each month) did not differ significantly between the San Francisco and Los Angeles cohorts (by the Kolomgorov-Smirnoff test: D = 0.25; *P* = .85, for a difference in the distribution of voucher redemption by month).

### Transportability Analysis

We weighted the concurrently sampled San Francisco group (SF2; n = 157) to demographically match the Los Angeles group (n = 155) (eTable 7 in the Supplement). We observed that if the San Francisco SF2 group were weighted to match the Los Angeles group in observed covariates, the weighted SF2 group would have an increase of 0.53 fruit and vegetable cup-equivalents per day (95% CI, 0.27-0.79 cup-equivalents; *P* = .03) vs the observed Los Angeles outcome of 0.64 cup-equivalents per day (95% CI, 0.41-0.88 cup-equivalents; *P* < .001), and a secondary outcome of an improvement of 6.8 in HEI score (95% CI, 3.5-10.0; *P* = .02) vs the observed Los Angeles outcome of 6.7 (95% CI, 5.6-8.4; *P* < .001). In other words, the observed demographic features rather than unmeasured factors could explain most of the between-site differences in outcomes. We observed that the interaction term between income and location was not significant (estimate of change in fruit and vegetable consumption, β = 0.0003 cup-equivalents; 95% CI, −0.0002 to 0.0009; estimate of change in HEI score, β = 0.0004; 95% CI, −0.0056 to 0.0064), suggesting that simple differences in the mean values of income were not sufficient to explain the difference across locations; the full distributional differences, accounted for in transportability analysis, were necessary for the explanation (eAppendix in the Supplement).

**Figure 2.**
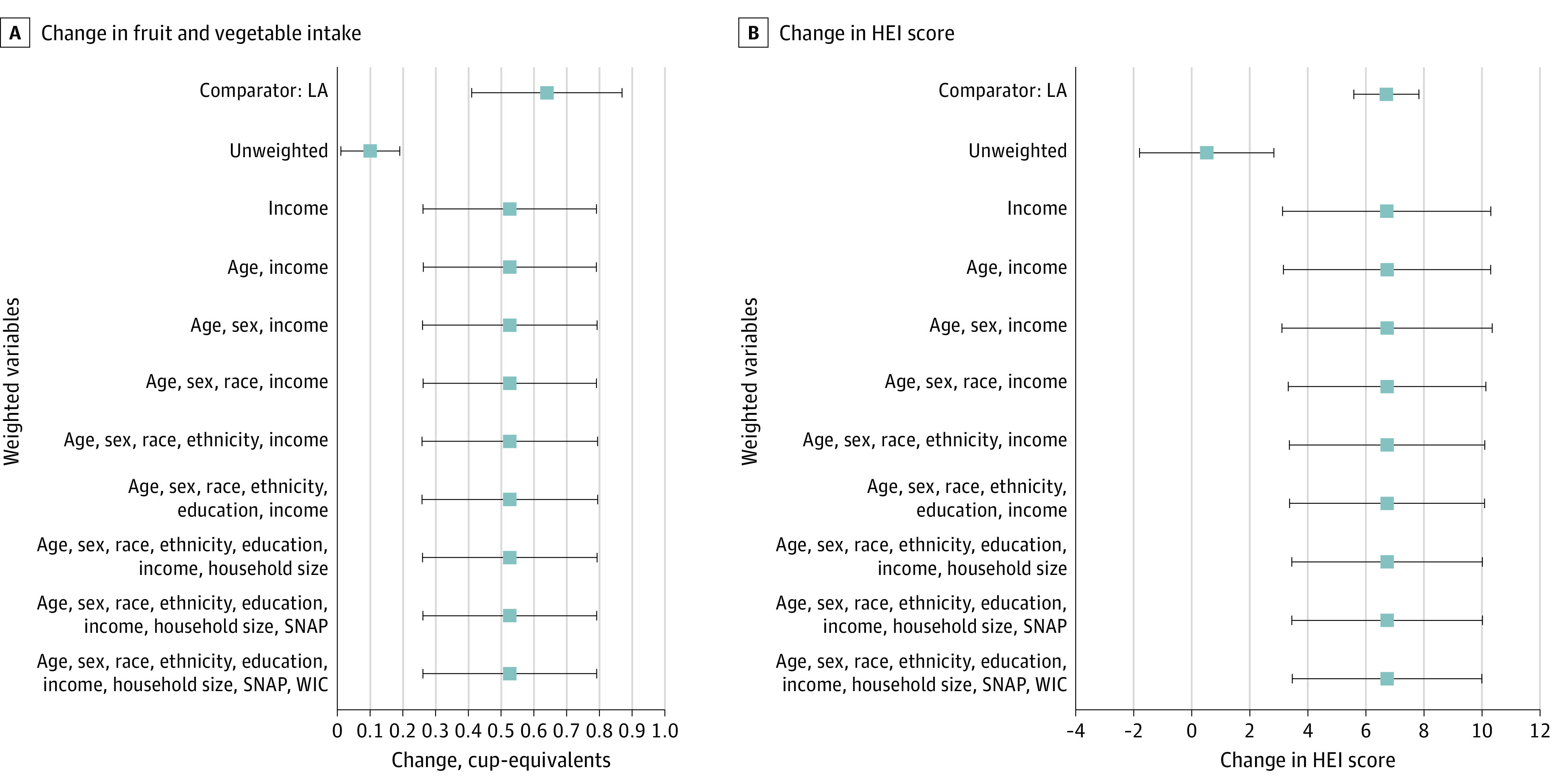
Unweighted and Weighted Outcomes Among the Second San Francisco Subpopulation Evaluated Concurrently With the Los Angeles Population With income alone as the variable with which to perform the weighting, the 95% CIs on the outcomes overlapped between the San Francisco and the Los Angeles groups, and minimally changed when other demographic variables were added to the weighting process. Error bars indicate 95% CIs. HEI indicates Healthy Eating Index; LA, Los Angeles; SNAP, Supplemental Nutrition Assistance Program; WIC, Women, Infants and Children.

In further analysis, we observed that without income included in the weighted analysis, the San Francisco SF2 group could not be reweighted to have its weighted outcome overlap with the outcome in Los Angeles; with income alone as the variable with which to perform the weighting, the 95% CIs on the outcomes overlapped between the San Francisco SF2 and the Los Angeles groups, and minimally changed when other demographic variables were added to the weighting process (Figure 2). Additional analyses did not reveal that exposure of the SF2 group to a sugar-sweetened beverage tax could explain variations among sites in the outcomes (eTables 8, 9, and 10 in the Supplement).

## Discussion

In this study of community-based voucher programs in 2 cities, use of vouchers was associated with increased fruit and vegetable intake overall. However, the vouchers had a weaker association with fruit and vegetable intake and a composite nutrition index (HEI score) in San Francisco than in Los Angeles, despite being accepted at more stores in San Francisco (29 in San Francisco vs 5 in Los Angeles). The voucher redemption rate did not explain the difference, but the lower income of the Los Angeles population did. One potential reason for this difference, consistent with food security literature,^16^ is that people with higher income purchase an amount closer to their goal for how much fruits and vegetables to consume before they receive vouchers. Hence, when they receive vouchers, the voucher money may be used in place of out-of-pocket funds (substitution of payment source), not resulting in a substantive increase in fruit and vegetable consumption overall. For lower income participants, in contrast, the extra value of the vouchers may likely increase total fruit and vegetable purchases (supplementing out-of-pocket funds rather than being a substitute for out-of-pocket funds). Notably, data from the Bureau of Economic Analysis suggest that the regional price parity for goods including food (the ratio of local price to national average, times 100) in San Francisco’s metro area is 114.7, vs the less expensive Los Angeles metro area at 105.7, indicating the same voucher may enable greater purchasing power in Los Angeles.^17^

As funders across the US consider supporting voucher programs,^18^ our data suggest that ensuring program benefits reach the lowest-income populations may be associated with higher per dollar outcomes on fruit and vegetable consumption. There appears to be heterogeneity even among the low-income populations that qualify for voucher programs. Programs often target a full subgroup of people below a given income threshold, but our findings would suggest disproportionate targeting for those with the lowest incomes. One way to effectively implement the targeting is by using a flat benefit rate (as done in this study) rather than a rate that scales to income; a flat rate is inherently worth relatively more for lower income people and is simpler to administer. The $20 per month for 6 months amounted to 2% of median income during the 6-month period among our study population. Low-income populations generally spend around 15% of their income on food.^18^ Hence, even voucher programs with small monetary amounts may provide increased support for fruit and vegetable consumption in the context of poverty.

We note that transportability analysis may be helpful to determine how factors that are measured (such as demographic characteristics and SNAP participation [and the chance of participants having received fruit/vegetable education through SNAP]) vs factors that are unmeasured (such as exact purchases, neighborhood or vendor characteristics, and food prices) may explain differences in the outcomes of interventions among cohorts; not all factors need to be measured for the transportability analysis to determine if the measured subset can explain differences in the outcome, which is a strength of the method.

### Limitations

This study has limitations. First, the study was based on a pre-post analysis without a control group not receiving vouchers. Secular trends in consumption may vary among different populations. Second, the transportability analysis may have been confounded by an unmeasured variable associated with income, the outcome variables, and site differences. Alternatively, and more likely, income may have been associated with both site differences and outcome variables in complex ways between the 2 study populations, such as by affecting how people manage their overall food budget, shop at lower priced stores, or purchase cheaper produce, which may be more available in lower-income Los Angeles neighborhoods. Third, our study was based on 24-hour dietary recalls, which are subject to recall and social acceptability biases that might inflate estimates because participants may wish to please the investigative staff by reporting higher fruit and vegetable intake after voucher receipt. Such mismeasurement, however, should not be differential between the study sites. In addition, our results may not generalize well to groups outside of the studied populations, which were largely of low-income, English-speaking individuals living in a single-member household.

## Conclusions

The results of this study support an association between receipt of fruit and vegetable vouchers and improvement of fruit and vegetable intake and overall dietary intake in low-income households. The results also suggest that the populations with the lowest incomes may derive more substantial benefit than those with higher incomes. We note that transportability analysis may be helpful to determine what factors may be associated with differences in the outcomes of interventions among cohorts.

## Notes

Supplement. **eAppendix.** **eTable 1.** Descriptive Statistics of the Study Participants, With the San Francisco Population Further Subdivided Between Earlier Versus Later Enrollment Groups **eTable 2.** Primary and Secondary Outcomes by Study Site, Disaggregating the San Francisco Population Into First (SF1) and Second (SF2) Groups, Where the Second Group Was Studied Concurrently With the Los Angeles Population **eTable 3.** Fully-adjusted Model Comparing the Concurrently-Sampled San Francisco (SF2, N = 157) and Los Angeles (N = 155) Populations, Adjusted for Demographics, Revealing the Voucher Was More Effective in Los Angeles Than in San Francisco in Terms of the Primary Outcome of Increase in Fruit and Vegetable Intake Between Months 0 and 6 **eTable 4.** Fully-adjusted Model Comparing the Concurrently-Sampled San Francisco (SF2, N = 157) and Los Angeles (N = 155) Populations, Adjusted for Demographics, Revealing the Voucher Was More Effective in Los Angeles Than in San Francisco in Terms of the Secondary Outcome of Healthy Eating Index (HEI) Score Improvement Between Months 0 and 6 **eTable 5.** HEI Subcomponents by Geographic Location and Time Period **eTable 6.** Regression Among the Concurrently Sampled San Francisco (SF2, N = 157) and Los Angeles (N = 155) Populations, Showing Interaction Term Between Voucher Redemption Rate and Primary Outcome of Fruit and Vegetable Intake After Adjustment for Demographics **eTable 7.** Pre and post-weighting Match Statistics Between the Second San Francisco Subpopulation (SF2, N = 157, the Population Measured Concurrently With Los Angeles) and the Unweighted (Observed) Los Angeles Population (N = 155) **eTable 8.** Sugar-Sweetened Beverage Intake by Study Site, Disaggregating the San Francisco Population Into First (SF1) and Second (SF2) Groups, Where the Second Group Was Studied Concurrently With the Los Angeles Population **eTable 9.** Regression Among the Concurrently Sampled San Francisco (SF2, N = 157) and Los Angeles (N = 155) Populations, Showing Interaction Term Between Sugar-Sweetened Beverage (SSB) Consumption (8 fl oz servings) and Primary Outcome of Fruit and Vegetable Intake After Adjustment for Demographics **eTable 10.** Regression Among the Concurrently Sampled San Francisco (SF2, N = 157) and Los Angeles (N = 155) Populations, Showing Interaction Term Between Sugar-Sweetened Beverage (SSB) Consumption (8 fl oz servings) and Secondary Outcome of Healthy Eating Index (HEI) Intake After Adjustment for Demographics
