# Supplementary material for: Comparison of Fruit and Vegetable Intake Among Urban Low-Income US Adults Receiving a Produce Voucher in 2 Cities
Source: JAMA Netw Open. 2021 Mar 22;4(3):e211757. doi: 10.1001/jamanetworkopen.2021.1757 (PMC7985725; doi:10.1001/jamanetworkopen.2021.1757)
Supplement: Supplement. — eAppendix. eTable 1. Descriptive Statistics of the Study Participants, With the San Francisco Population Further Subdivided Between Earlier Versus Later Enrollment Groups eTable 2. Primary and Secondary Outcomes by Study Site, Disaggregating the San Francisco Population Into First (SF1) and Second (SF2) Groups, Where the Second Group Was Studied Concurrently With the Los Angeles Population eTable 3. Fully-adjusted Model Comparing the Concurrently-Sampled San Francisco (SF2, N = 157) and Los Angeles (N = 155) Populations, Adjusted for Demographics, Revealing the Voucher Was More Effective in Los Angeles Than in San Francisco in Terms of the Primary Outcome of Increase in Fruit and Vegetable Intake Between Months 0 and 6 eTable 4. Fully-adjusted Model Comparing the Concurrently-Sampled San Francisco (SF2, N = 157) and Los Angeles (N = 155) Populations, Adjusted for Demographics, Revealing the Voucher Was More Effective in Los Angeles Than in San Francisco in Terms of the Secondary Outcome of Healthy Eating Index (HEI) Score Improvement Between Months 0 and 6 eTable 5. HEI Subcomponents by Geographic Location and Time Period eTable 6. Regression Among the Concurrently Sampled San Francisco (SF2, N = 157) and Los Angeles (N = 155) Populations, Showing Interaction Term Between Voucher Redemption Rate and Primary Outcome of Fruit and Vegetable Intake After Adjustment for Demographics eTable 7. Pre and post-weighting Match Statistics Between the Second San Francisco Subpopulation (SF2, N = 157, the Population Measured Concurrently With Los Angeles) and the Unweighted (Observed) Los Angeles Population (N = 155) eTable 8. Sugar-Sweetened Beverage Intake by Study Site, Disaggregating the San Francisco Population Into First (SF1) and Second (SF2) Groups, Where the Second Group Was Studied Concurrently With the Los Angeles Population eTable 9. Regression Among the Concurrently Sampled San Francisco (SF2, N = 157) and Los Angeles (N = 155) Populations, Showing Interaction Term B [file jamanetwopen-e211757-s001.pdf]

# Supplemental Online Content

Basu S, Akers M, Berkowitz SA, Josey K, Schillinger D, Seligman H. Comparison of fruit and vegetable intake among urban low-income US adults receiving a produce voucher in 2 cities. *JAMA Netw Open*. 2021;4(3):e211757. doi:10.1001/jamanetworkopen.2021.1757

**eAppendix.**
**eTable 1.** Descriptive statistics of the Study Participants, With the San Francisco Population Further Subdivided Between Earlier Versus Later Enrollment Groups
**eTable 2.** Primary and Secondary Outcomes by Study Site, Disaggregating the San Francisco Population Into First (SF1) and Second (SF2) Groups, Where the Second Group Was Studied Concurrently With the Los Angeles Population
**eTable 3.** Fully-adjusted Model Comparing the Concurrently-Sampled San Francisco (SF2, N = 157) and Los Angeles (N = 155) Populations, Adjusted for Demographics, Revealing the Voucher Was More Effective in Los Angeles Than in San Francisco in Terms of the Primary Outcome of Increase in Fruit and Vegetable Intake Between Months 0 and 6
**eTable 4.** Fully-adjusted Model Comparing the Concurrently-Sampled San Francisco (SF2, N = 157) and Los Angeles (N = 155) Populations, Adjusted for Demographics, Revealing the Voucher Was More Effective in Los Angeles Than in San Francisco in Terms of the Secondary Outcome of Healthy Eating Index (HEI) Score Improvement Between Months 0 and 6
**eTable 5.** HEI Subcomponents by Geographic Location and Time Period
**eTable 6.** Regression Among the Concurrently Sampled San Francisco (SF2, N = 157) and Los Angeles (N = 155) Populations, Showing Interaction Term Between Voucher Redemption Rate and Primary Outcome of Fruit and Vegetable Intake After Adjustment for Demographics
**eTable 7.** Pre and post-weighting Match Statistics Between the Second San Francisco Subpopulation (SF2, N = 157, the Population Measured Concurrently With Los Angeles) and the Unweighted (Observed) Los Angeles Population (N = 155)
**eTable 8.** Sugar-Sweetened Beverage Intake by Study Site, Disaggregating the San Francisco Population Into First (SF1) and Second (SF2) Groups, Where the Second Group Was Studied Concurrently With the Los Angeles Population
**eTable 9.** Regression Among the Concurrently Sampled San Francisco (SF2, N = 157) and Los Angeles (N = 155) Populations, Showing Interaction Term Between Sugar-Sweetened Beverage (SSB) Consumption (8 fl oz servings) and Primary Outcome of Fruit and Vegetable Intake After Adjustment for Demographics
**eTable 10.** Regression Among the Concurrently Sampled San Francisco (SF2, N = 157) and Los Angeles (N = 155) Populations, Showing Interaction Term Between Sugar-Sweetened Beverage (SSB) Consumption (8 fl oz servings) and Secondary Outcome of Healthy Eating Index (HEI) Intake After Adjustment for Demographics

© 2021 Basu S et al. *JAMA Network Open*.

This supplemental material has been provided by the authors to give readers additional information about their work.

© 2021 Basu S et al. *JAMA Network Open*.

**eAppendix**

**Transportability methods.**

Transportability methods seek to understand how and why estimates of the average treatment effect of an intervention may vary across study populations, given that effect modifiers (effect-modifying covariates) may differ among study populations.

In a regression analysis, the value that is estimated is the conditional average treatment effect (conditioned on the covariates), whereas in randomized trials and in causal inference, we estimate a marginal average treatment effect (i.e., we marginalize out the other covariates). The weighting estimator, in a sense, integrates the conditional average treatment effect over the empirical distribution of the covariates to get an estimate of the marginal average treatment effect. In this particular case, we integrate over the empirical distribution observed in the target population (Los Angeles).

In this study, we use the transportability method proposed by Josey and colleagues,[14] who use an entropy balancing approach that seeks to balance entire distributions of covariates (potential effect modifiers) across populations in a study, weighting one population to be more similar to another, to understand how much different covariates may explain differences in estimated average treatment effect. Entropy balancing is a preferred approach to transportability estimation because it has been shown to be doubly-robust (enabling either the weight-estimating equation or the effect-estimating equation to be misspecified without introducing bias) and focuses on an entire distribution of covariates among participants rather than simply the mean values of a group.

The transportability method depends on the definition of the group average treatment effect as:

$$\tau = \frac{1}{n_0} \sum_{\{i:S_i=0\}} [Y_i(1) - Y_i(0)]$$

where the study's population group is $i = 1, 2, \ldots, n$, $S_i \in \{0, 1\}$ denotes the control and intervention observations, such that $n_1 = \sum_{i=1}^{n} S_i, n_0 = \sum_{i=1}^{n} (1 - S_i), Y_i \in \mathfrak{R}$ is the outcome where $Y_i(0)$ and $Y_i(1)$ refer to each unit's outcomes when variable $Z_i = 1$ and $Z_i = 0$, respectively, and $Z_i \in \{0, 1\}$ denote control versus treatment conditions.

© 2021 Basu S et al. *JAMA Network Open*.

The entropy balancing approach to transportability estimation is an extension of the methods of moments approach proposed by Signorovitch and colleagues,[21] where given $x_i \in x$ of measured covariates, the balance function used to model the moments for $S_i$ , $Y_i$, and $Z_i$ is defined as $\tilde{X}_i = (2Z_i - 1, X_i)$and the target covariate distribution $\hat{\theta}_0 = E[X_i | S_i = 0]$ with $\tilde{\theta}_0 = (0, \hat{\theta}_0)$. The method of moments estimator is defined by the Lagrangian dual problem:

$$\hat{\lambda} = argmax_{\lambda \in \mathfrak{R}^{m+1}} \sum_{\{i:S_i=1\}} [-exp(\tilde{X}_i{}^T \lambda) - \tilde{\theta}_0{}^T \lambda],$$

which is used to estimate the sampling weights:

$$\hat{\gamma}_i^{MOM} = exp\,(-\tilde{X}_i{}^T \hat{\lambda}) \text{for all } i \in \{ i: S_i = 1\}.$$

In the entropy balancing approach proposed by Josey and colleagues,[14] the Langragian dual problem is instead defined as:

$$\hat{\lambda}_0 = argmax_{\lambda \in \mathfrak{R}^m} \textstyle\sum_{\{i:S_i=1\}} [-exp(-(1 - Z_i)X_i{}^T \lambda) - \hat{\theta}_0{}^T \lambda] \quad \text{and}$$

$$\hat{\lambda}_1 = argmax_{\lambda \in \mathfrak{R}^m} \sum_{\{i:S_i=1\}} [-exp(-Z_i X_i{}^T \lambda) - \hat{\theta}_0{}^T \lambda]$$

From which the sampling weights are computed as:

$$\hat{\gamma}_i^{EB} = exp\,[-(1 - Z_i)X_i{}^T \hat{\lambda}_0 - Z_i X_i{}^T \hat{\lambda}_1 ] \text{for all } i \in \{ i: S_i = 1\}.$$

The three main assumptions to consider when applying the transportability methods are (i) mean exchangeability, which means that the mean of potential outcomes are exchangeable among the populations, conditional on their covariates; (ii) sampling positivity, which means that the probability of participating in the study is given the covariates is not near zero or one; and (ii) strongly ignorable treatment assignment, which means that all participants could have the same potential outcomes regardless of their current treatment status. The statistical code for this computation is provided online with the overall code to reproduce results of this study, at https://github.com/sanjaybasu/vouchertransportability/.

**Additional analyses related to sugar-sweetened beverage consumption.**

A penny-per-ounce sugar-sweetened beverage tax was implemented in San Francisco in January 2018, while no such tax existed in Los Angeles.[22] This means that participants in the SF1 group and Los Angeles group were not subject to a tax, but those in SF2 were. As such, it is possible that in San Francisco, the sugary beverage tax might alter the effects of a fruits and vegetable voucher with respect to dietary quality, an effect that would also extend to the

© 2021 Basu S et al. *JAMA Network Open*.

comparison between the concurrent San Francisco SF2 and Los Angeles groups. However, the fact that we observed only small and nearly identical changes in dietary quality for the two waves of the program in San Francisco over this timeframe (comparing pre- and post-tax periods) makes it unlikely that the tax had a significant interaction with the voucher program. Nevertheless, we formally compared the differences in sugar-sweetened beverage consumption among each subgroup of participants (**eTable 7**), finding no significant reduction in sugar-sweetened beverage consumption after the tax, and no difference between the concurrently-studied San Francisco (SF2) and Los Angeles groups. We examined whether there was any interaction between change in sugar-sweetened beverage consumption and location for the two outcomes of fruit and vegetable intake (**eTable 8**) and HEI score (**eTable 9**), and found none.

As shown in **eTable 8**, including sugar-sweetened beverage consumption variables -- baseline sugar-sweetened beverage consumption, changes in sugar-sweetened beverage consumption, and interaction between changes in sugar-sweetened beverage consumption and site--did not alter the main effect of fruit and vegetable consumption, such that any reduction in sugar-sweetened beverage consumption that we might have observed due to the tax did not influence this outcome. As shown In **eTable 9**, the sugar-sweetened beverage consumption variables do not alter the main effect regarding Healthy Eating Index but the change in sugar-sweetened beverage consumption was associated with HEI scores. However, the changes in sugar-sweetened beverage consumption observed by site did not explain the site-related difference in change in HEI scores (interaction variable), such that any reduction in sugar-sweetened beverage consumption that we might have observed due to the tax may have been small or the study lacked power to detect it.

© 2021 Basu S et al. *JAMA Network Open*.

**eTable 1.** Descriptive statistics of the study participants, with the San Francisco population further subdivided between earlier versus later enrollment groups.

| Characteristic | Los Angeles | San Francisco, first group studied earlier than Los Angeles | San Francisco, second group studied concurrently with Los Angeles | P-value for difference |
|---|---|---|---|---|
| n | 155 | 359 | 157 | |
| Age, yrs (median [IQR]) | 58.00 [46.50, 67.00] | 54.00 [41.50, 61.00] | 55.00 [45.00, 64.00] | 0.001 |
| Female (%) | 90 (58.1) | 232 (64.6) | 92 (58.6) | 0.212 |
| Black (%) | 71 (45.8) | 104 (29.0) | 32 (20.4) | <0.001 |
| Hispanic (%) | 52 (33.5) | 56 (15.6) | 24 (15.3) | <0.001 |
| Education level (%) | | | | 0.001 |
| Never attended | 1 ( 0.6) | 1 ( 0.3) | 0 ( 0.0) | |
| Elementary or Middle School | 9 ( 5.8) | 6 ( 1.7) | 1 ( 0.6) | |
| Some high school | 19 (12.3) | 22 ( 6.1) | 11 ( 7.0) | |
| High school graduate | 37 (23.9) | 66 (18.4) | 21 (13.4) | |
| Some college/tech school | 59 (38.1) | 139 (38.7) | 72 (45.9) | |
| College graduate | 26 (16.8) | 102 (28.4) | 43 (27.4) | |
| Other | 4 ( 2.6) | 23 ( 6.4) | 9 ( 5.7) | |
| Household income, monthly $ (median [IQR]) | 916.67 [668.34, 1200.00] | 1000.00 [800.23, 1400.00] | 1000.00 [895.00, 1500.00] | 0.004 |
| Household size, people (median [IQR]) | 1.00 [1.00, 2.00] | 1.00 [1.00, 2.00] | 1.00 [1.00, 2.00] | 0.903 |
| SNAP participant (%) | 58 (37.4) | 99 (27.6) | 46 (29.3) | 0.08 |
| WIC participant (%) | 3 ( 1.9) | 11 ( 3.1) | 4 ( 2.5) | 0.762 |

© 2021 Basu S et al. *JAMA Network Open*.

**eTable 2.** Primary and secondary outcomes by study site, disaggregating the San Francisco population into first (SF1) and second (SF2) groups, where the second group was studied concurrently with the Los Angeles population.

| Outcome | Month 0, baseline (IQR) | Month 6, during voucher (IQR) | Within-person change, month 0 to month 6 (95% CI) | P value for within-person change |
|---|---|---|---|---|
| **Fruit and vegetable intake (cup-equivalents)** | | | | |
| Overall population (N = 671) | 1.10 (0.47, 1.55) | 1.33 (0.61, 1.75) | 0.22 (0.14, 0.31) | <0.001 |
| San Francisco first group (N = 359), SF1 | 1.07 (0.50, 1.52) | 1.17 (0.57, 1.58) | 0.09 (-0.29, 0.51) | 0.058 |
| San Francisco second group (N = 157), SF2 | 1.30 (0.59, 1.78) | 1.46 (0.62, 1.95) | 0.14 (-0.51, 0.58) | 0.205 |
| Los Angeles (N = 155) | 0.97 (0.31, 1.34) | 1.58 (0.71, 2.12) | 0.64 (0.41, 0.88) | <0.001 |
| **Healthy Eating Index (0-100)** | | | | |
| Overall population (N = 671) | 61.9 (52.4, 71.3) | 64.2 (54.7, 73.8) | 2.0 (1.0, 3.0) | <0.001 |
| San Francisco first group (N = 359), SF1 | 62.5 (53.3, 71.3) | 63.4 (54.2, 72.8) | 0.6 (-0.5, 1.8) | 0.292 |
| San Francisco second group (N = 157), SF2 | 62.9 (53.9, 71.3) | 64.3 (53.7, 74.4) | 0.5 (-1.8, 2.8) | 0.647 |
| Los Angeles (N = 155) | 59.5 (49.6, 70.2) | 66.1 (57.9, 75.8) | 6.8 (4.3, 9.2) | <0.001 |

© 2021 Basu S et al. *JAMA Network Open*.

**eTable 3.** Fully-adjusted model comparing the concurrently-sampled San Francisco (SF2, N = 157) and Los Angeles (N = 155) populations, adjusted for demographics, revealing the voucher was more effective in Los Angeles than in San Francisco in terms of the primary outcome of increase in fruit and vegetable intake between months 0 and 6.

| Covariate | Regression coefficient (beta estimate) | Standard error | P-value |
|---|---|---|---|
| Age, yrs | -0.005 | 0.007 | 0.503 |
| Sex, female | -0.134 | 0.173 | 0.440 |
| Race, Black | -0.081 | 0.198 | 0.682 |
| Ethnicity, Hispanic | -0.019 | 0.225 | 0.931 |
| Elementary or Middle School | 0.658 | 1.448 | 0.650 |
| Some high school | 0.969 | 1.424 | 0.497 |
| High school graduate | 1.221 | 1.404 | 0.386 |
| Some college/tech school | 0.665 | 1.400 | 0.635 |
| College graduate | 1.012 | 1.410 | 0.474 |
| Household income, monthly $ | 0.000 | 0.000 | 0.773 |
| Household size, people | -0.025 | 0.083 | 0.758 |
| SNAP participant (%) | 0.152 | 0.183 | 0.408 |
| WIC participant (%) | -0.224 | 0.586 | 0.702 |
| San Francisco location, relative to Los Angeles | -0.500 | 0.188 | 0.008 |

Adjusted R-squared: 0.03084

© 2021 Basu S et al. *JAMA Network Open*.

**eTable 4.** Fully-adjusted model comparing the concurrently-sampled San Francisco (SF2, N = 157) and Los Angeles (N = 155) populations, adjusted for demographics, revealing the voucher was more effective in Los Angeles than in San Francisco in terms of the secondary outcome of Healthy Eating Index (HEI) score improvement between months 0 and 6.

| Covariate | Regression coefficient (beta estimate) | Standard error | P-value |
|---|---|---|---|
| Age, yrs | 0.086 | 0.071 | 0.226 |
| Sex, female | 0.340 | 1.817 | 0.852 |
| Race, Black | -3.836 | 2.079 | 0.066 |
| Ethnicity, Hispanic | -1.395 | 2.362 | 0.555 |
| Elementary or Middle School | 16.328 | 15.176 | 0.283 |
| Some high school | 16.986 | 14.925 | 0.256 |
| High school graduate | 18.908 | 14.721 | 0.200 |
| Some college/tech school | 16.233 | 14.680 | 0.270 |
| College graduate | 13.462 | 14.786 | 0.363 |
| Household income, monthly $ | -0.001 | 0.001 | 0.359 |
| Household size, people | -1.461 | 0.865 | 0.093 |
| SNAP participant | 0.514 | 1.918 | 0.789 |
| WIC participant | -8.665 | 6.141 | 0.160 |
| San Francisco location, relative to Los Angeles | -6.513 | 1.972 | 0.001 |

Adjusted R-squared: 0.06751

© 2021 Basu S et al. *JAMA Network Open*.

**eTable 5.** HEI subcomponents by geographic location and time period.

| Outcome | Los Angeles (N = 155 for month 0, N = 139 for month 6) | | | San Francisco (N = 516 for month 0, N = 465 for month 6) | | |
|---|---|---|---|---|---|---|
| **Healthy Eating Index subcomponent (range: poor score to good score)** | **Month 0, baseline (SE)** | **Month 6, during voucher (SE)** | **Standardized mean difference** | **Month 0, baseline (SE)** | **Month 6, during voucher (SE)** | **Standardized mean difference** |
| Total fruits (0-5) | 3.40 (2.07) | 4.20 (1.61) | 0.43 | 3.72 (1.77) | 3.90 (1.72) | 0.10 |
| Whole fruits (0-5) | 3.30 (2.18) | 4.15 (1.75) | 0.43 | 3.84 (1.89) | 3.93 (1.86) | 0.05 |
| Total vegetables (0-5) | 4.24 (1.37) | 4.51 (1.23) | 0.21 | 4.43 (1.15) | 4.51 (1.06) | 0.07 |
| Green beans (0-5) | 2.82 (2.41) | 3.59 (2.14) | 0.34 | 3.43 (2.13) | 3.59 (2.10) | 0.07 |
| Whole grains (0-5) | 3.89 (3.89) | 4.76 (3.99) | 0.22 | 3.98 (3.67) | 4.31 (3.75) | 0.09 |
| Dairy (0-10) | 4.46 (3.31) | 4.83 (3.31) | 0.11 | 5.56 (3.18) | 5.54 (3.16) | 0.01 |
| Total protein (0-5) | 4.41 (1.15) | 4.58 (1.06) | 0.15 | 4.54 (0.96) | 4.52 (1.00) | 0.02 |
| Seafood/plant protein (0-5) | 2.05 (2.26) | 2.06 (2.21) | 0.00 | 2.26 (2.08) | 2.09 (2.07) | 0.08 |
| Fatty acids (10-0) | 5.15 (3.42) | 5.23 (3.74) | 0.02 | 5.15 (3.44) | 5.09 (3.45) | 0.02 |
| Refined grains (10-0) | 7.42 (3.24) | 8.41 (2.81) | 0.33 | 7.56 (2.99) | 7.63 (3.01) | 0.02 |
| Sodium (10-0) | 1.82 (3.09) | 2.66 (3.86) | 0.24 | 1.58 (2.96) | 1.64 (3.03) | 0.02 |
| Added sugars (10-0) | 6.84 (3.57) | 7.43 (3.23) | 0.18 | 7.03 (3.20) | 7.42 (3.01) | 0.12 |
| Saturated fats (10-0) | 9.69 (1.17) | 9.70 (1.01) | 0.01 | 9.57 (1.27) | 9.50 (1.41) | 0.05 |

© 2021 Basu S et al. *JAMA Network Open*.

**eTable 6.** Regression among the concurrently-sampled San Francisco (SF2, N = 157) and Los Angeles (N = 155) populations, showing interaction term between voucher redemption rate and primary outcome of fruit and vegetable intake after adjustment for demographics.

| Covariate | Regression coefficient (beta estimate) | Standard error | P-value |
|---|---|---|---|
| Age, yrs | -0.006 | 0.007 | 0.411 |
| Sex, female | 0.015 | 0.014 | 0.272 |
| Race, Black | -0.102 | 0.199 | 0.610 |
| Ethnicity, Hispanic | -0.063 | 0.226 | 0.781 |
| Elementary or Middle School | 0.528 | 1.438 | 0.714 |
| Some high school | 0.707 | 1.411 | 0.617 |
| High school graduate | 1.016 | 1.393 | 0.466 |
| Some college/tech school | 0.455 | 1.391 | 0.744 |
| College graduate | 0.799 | 1.400 | 0.569 |
| Household income, monthly $ | 0.000 | 0.000 | 0.828 |
| Household size, people | -0.060 | 0.082 | 0.464 |
| SNAP participant | 0.202 | 0.185 | 0.275 |
| WIC participant | -0.275 | 0.583 | 0.638 |
| San Francisco location, relative to Los Angeles | 0.423 | 0.642 | 0.511 |
| Voucher redemption rate | 0.800 | 0.550 | 0.147 |
| Interaction between San Francisco location and voucher redemption rate | -1.191 | 0.805 | 0.140 |

Adjusted R-squared: 0.02902

© 2021 Basu S et al. *JAMA Network Open*.

**eTable 7.** Pre and post-weighting match statistics between the second San Francisco subpopulation (SF2, N = 157, the population measured concurrently with Los Angeles) and the unweighted (observed) Los Angeles population (N = 155).

| Characteristic | San Francisco, unweighted mean (SD), N = 157 | San Francisco population, weighted mean (SD), N = 157 | Los Angeles population, unweighted mean (SD), N = 155 |
|---|---|---|---|
| Age, yrs | 53.9 (14.2) | 56.3 (13.3) | 58.3 (13.4) |
| Sex, proportion female | 0.6 | 0.6 | 0.6 |
| Black, proportion | 0.2 | 0.5 | 0.5 |
| Hispanic, proportion | 0.1 | 0.3 | 0.3 |
| Education, yrs after 5th grade | 10.2 (21.5) | 7.1 (15.5) | 7.1 (15.5) |
| Household monthly income, mean $ [note: eTable 1 has median] | 1231.8 (701.3) | 939.4 (531.8) | 1166.1 (605.0) |
| People in household | 1.6 (1) | 1.7 (1.3) | 1.8 (1.3) |
| Proportion SNAP participants | 0.3 | 0.4 | 0.3 |
| Proportion WIC participants | 0 | 0 | 0 |

© 2021 Basu S et al. *JAMA Network Open*.

**eTable 8.** Sugar-sweetened beverage intake by study site, disaggregating the San Francisco population into first (SF1) and second (SF2) groups, where the second group was studied concurrently with the Los Angeles population.

| Outcome | Month 0, baseline (IQR) | Month 6, during voucher (IQR) | Within-person change, month 0 to month 6 (95% CI) | P value for within-person change |
|---|---|---|---|---|
| **Sugar-sweetened beverage intake (servings, 8 fl oz)** | | | | |
| Overall population (N = 671) | 0.59 (0.00, 0.75) | 0.50 (0.00, 0.63) | -0.06 (-0.14, 0.02) | 0.142 |
| San Francisco first group (N = 359), SF1 | 0.52 (0.0, 0.64) | 0.51 (0.00, 0.62) | 0.00 (-0.09, 0.09) | 0.970 |
| San Francisco second group (N = 157), SF2 | 0.59 (0.00, 0.75) | 0.40 (0.00, 0.50) | -0.13 (-0.32, 0.05) | 0.151 |
| Los Angeles (N = 155) | 0.75 (0.00, 1.06) | 0.58 (0.00, 0.75) | -0.13 (-0.32, 0.07) | 0.194 |

© 2021 Basu S et al. *JAMA Network Open*.

**eTable 9.** Regression among the concurrently-sampled San Francisco (SF2, N = 157) and Los Angeles (N = 155) populations, showing interaction term between sugar-sweetened beverage (SSB) consumption (8 fl oz servings) and primary outcome of fruit and vegetable intake after adjustment for demographics.

| Covariate | Regression coefficient (beta estimate) | Standard error | P-value |
|---|---|---|---|
| Age, yrs | -0.005 | 0.007 | 0.464 |
| Sex, female | 0.016 | 0.014 | 0.251 |
| Race, Black | -0.108 | 0.198 | 0.587 |
| Ethnicity, Hispanic | -0.025 | 0.226 | 0.913 |
| Elementary or Middle | 0.544 | 1.439 | 0.706 |
| School | 0.787 | 1.412 | 0.578 |
| Some high school | 1.047 | 1.396 | 0.454 |
| High school graduate | 0.509 | 1.393 | 0.715 |
| Some college/tech school | 0.864 | 1.402 | 0.538 |
| College graduate | 0.158 | 1.454 | 0.913 |
| Household income, monthly $ | 0.000 | 0.000 | 0.962 |
| Household size, people | -0.053 | 0.081 | 0.513 |
| SNAP participant | 0.089 | 0.184 | 0.630 |
| WIC participant | -0.241 | 0.591 | 0.684 |
| San Francisco location, relative to Los Angeles | -0.469 | 0.207 | 0.024 |
| SSB baseline consumption | -0.024 | 0.125 | 0.846 |
| SSB change in consumption, month 0 to month 6 | -0.203 | 0.130 | 0.120 |
| Interaction between San Francisco location and baseline SSB consumption | -0.061 | 0.177 | 0.729 |
| Interaction between San Francisco location and change in SSB consumption | 0.228 | 0.207 | 0.272 |

Adjusted R-squared: 0.03455

© 2021 Basu S et al. *JAMA Network Open*.

**eTable 10.** Regression among the concurrently-sampled San Francisco (SF2, N = 157) and Los Angeles (N = 155) populations, showing interaction term between sugar-sweetened beverage (SSB) consumption (8 fl oz servings) and secondary outcome of Healthy Eating Index (HEI) intake after adjustment for demographics.

| Covariate | Regression coefficient (beta estimate) | Standard error | P-value |
|---|---|---|---|
| Age, yrs | 0.063 | 0.042 | 0.134 |
| Sex, female | 0.681 | 1.097 | 0.535 |
| Race, Black | -1.486 | 1.197 | 0.215 |
| Ethnicity, Hispanic | -0.631 | 1.383 | 0.649 |
| Elementary or Middle | 11.778 | 9.485 | 0.215 |
| School | 10.718 | 9.299 | 0.250 |
| Some high school | 9.934 | 9.187 | 0.280 |
| High school graduate | 9.618 | 9.158 | 0.294 |
| Some college/tech school | 9.552 | 9.217 | 0.300 |
| College graduate | 9.033 | 9.431 | 0.339 |
| Household income, monthly $ | 0.000 | 0.001 | 0.894 |
| Household size, people | -0.861 | 0.552 | 0.119 |
| SNAP participant | -0.841 | 1.142 | 0.462 |
| WIC participant | -2.496 | 3.233 | 0.440 |
| San Francisco location, relative to Los Angeles | -6.452 | 1.788 | 0.000 |
| SSB baseline consumption | -0.264 | 1.128 | 0.815 |
| SSB change in consumption, month 0 to month 6 | -4.741 | 1.186 | 0.000 |
| Interaction between San Francisco location and baseline SSB consumption | 0.099 | 1.605 | 0.951 |
| Interaction between San Francisco location and change in SSB consumption | 2.713 | 1.873 | 0.148 |

Adjusted R-squared: 0.07823

© 2021 Basu S et al. *JAMA Network Open*.
